# Unplanned Extubation in Extremely Preterm Neonates: Incidence, Risk Factors, and Impact on Clinical Outcomes

**DOI:** 10.7759/cureus.73688

**Published:** 2024-11-14

**Authors:** Linda Ibrahim, Jihan Deghidy, Bilal Kanth, Habeebah Fazlullah, Apple Layug, Iqra Abid, Ashraf I Gad

**Affiliations:** 1 Women's Wellness and Research Center, Hamad Medical Corporation, Doha, QAT; 2 Women's Wellness and Research Center, Hamad Medical Corporation, doha, QAT; 3 Department of Respiratory Medicine, Sidra Medicine, Doha, QAT

**Keywords:** accidental extubation, extremely low birth weight, extremely preterm, infants, neonates, nicu, reintubation, unplanned extubation

## Abstract

Background

Unplanned extubation (UE) poses a significant safety risk to mechanically ventilated, preterm, and critically ill neonates in the neonatal intensive care unit (NICU).

Objective

The aim of this study was to evaluate the incidence of UE from January 2018 to December 2021, identify contributing risk factors, and compare the outcomes with a cohort of extremely preterm (EP) infants.

Methods

A retrospective study was conducted in the NICU at the Women’s Wellness and Research Center, Hamad Medical Corporation in Qatar. The study included 25 EP neonates who experienced UE events. The characteristics and outcomes of these infants were compared with a matched cohort of 75 EP infants without UE, selected using propensity score matching at a ratio of 1:3 to balance key baseline characteristics. This study was initiated in early 2018 following the introduction of a care improvement bundle that integrated various care practices and involved multiple healthcare staff.

Results

We recorded 25 UE events in our cohort of 507 EP neonates, totaling 5,668 invasive ventilation days. The incidence of UE events was 0.44 per 100 ventilation days over the four-year period, ranging from 0.60 in 2018 to 0.27 in 2020. UE occurred mainly during routine care activities (24%), due to agitation (20%), or during endotracheal tube manipulation (20%). Following a UE event, 64% of the neonates required positive pressure ventilation, and 88% were reintubated. Comparisons between the UE and non-UE groups revealed that UE was associated with significantly higher rates of bronchopulmonary dysplasia (BPD) (91.3% vs. 59.6%, p = 0.006), severe BPD (34.8% vs. 8.8%, p = 0.008), and increased postnatal steroid use (72.0% vs. 18.7%, p < 0.001). Neonates in the UE group had significantly longer hospital stays (127.0 days (IQR: 112.0-183.2) vs. 101.0 days (IQR: 90.0-139.5), p = 0.010), a higher median discharge postmenstrual age (42.8 weeks (IQR: 41.1-50.4) vs. 40.1 weeks (IQR: 37.3-46.6), p = 0.006), and a higher rate of receptive neurodevelopmental delays (50.0% vs. 19.5%, p = 0.009).

Conclusion

Neonates who experienced UE faced an increased risk of adverse respiratory and neurodevelopmental outcomes, including higher rates of BPD, increased postnatal steroid use, and longer NICU stays. This highlights the critical role of nursing care and continuous quality improvement efforts in the NICU to prevent UE.

## Introduction

Unplanned extubation (UE) refers to the unintentional dislodgement of an endotracheal tube (ETT) from a patient receiving invasive mechanical ventilation (MV) [1]. UE requiring reintubation is one of the most common adverse safety events in extremely preterm (EP) neonates [2,3].

The incidence of UE among neonates receiving MV ranges from 0.41 to 2.38 per 100 intubation days [1, 3], which is higher compared to other pediatric populations (0.61 per 100 intubation days) [4]. This increased rate is likely influenced by factors such as prolonged intubation duration and the unique challenges of neonatal care.

While there are limited large-scale studies of UE in pediatric populations, the associated morbidity is significant. Reports have documented cardiovascular collapse and even cardiac arrest in up to 20% of cases involving UE events. Additionally, reintubation rates for patients who experience UE range from 49% to 69% [5, 6]. Pediatric UE is also associated with increased ICU costs and length of stay [7, 8]. Several neonatal intensive care units (NICUs) have attempted to identify the risk factors for UE in neonates to help establish effective prevention strategies [3]. According to relevant studies, the risk factors can be classified into patient-related, intubation-related, and nursing-related factors. Patient-related factors include gestational age (GA), birth weight (BW), patient instability and agitation, duration of MV, and ventilator weaning [3,9,10]. Intubation-related factors comprise the duration of endotracheal intubation, fixation of the ETT, and the length of the ETT fixation [11,12]. Nursing-related factors include the nurse-patient ratio as well as nurses' workload, and the timing of work shifts (e.g., day, evening, or night shift), and nursing activities (e.g., suction, ETT refixation, body weight measurement, patient positioning) [1,9,13,14]. Le Blanc G et al. highlighted the significant impact of nursing overtime on UE occurrences in NICUs and its association with BPD in neonates born at less than 29 weeks who require reintubation [14]. Moreover, several NICUs have established the goal of achieving a UE rate of one or lower per 100 intubation days and have accordingly developed various preventive measures and interventions [15,16].

Various techniques and strategies are employed to lower the UE rate in NICUs [17]. Quality improvement (QI) studies have effectively used care bundles to decrease the occurrence of UE events in the NICU [18,19]. Besides, there is an association between compliance with regulated bundled fundamentals of care and a decrease in UE rates and related adverse events [20]. Carvalho F et al. emphasized the need for aggressive ventilator weaning and early extubation with regular evaluation of extubation readiness parameters as methods to decrease UE, along with using protocols for the early identification of neonates ready for extubation [21].

In January 2018, our unit implemented a care improvement bundle for intubated neonates in response to the previous year’s high rate of UEs, which was 2.31 per 100 ventilation days across the NICU. The goal was to reduce this rate by 50% within the following year. This care bundle involved comprehensive changes in care practices and included the collaboration of various healthcare professionals, such as nurses, physicians, respiratory therapists, and physical therapists [1,13,17,22].

Despite existing studies on UE in NICUs, there remains a notable gap in the literature regarding specific risk factors and the short- and long-term outcomes associated with UE, particularly following the implementation of care improvement bundles [17]. This gap is even more pronounced in Middle Eastern NICUs, where variations in healthcare funding, resource availability, and staff training potentially influence patient care and outcomes. Additionally, there is limited evidence comparing the clinical outcomes of EP neonates with and without UE, who are particularly vulnerable to these events.

Given these gaps, our study aimed to investigate the risk factors and outcomes associated with UE in a cohort of EP neonates. We hypothesized that UE increases the risk of both short- and long-term complications in this vulnerable population.

## Materials and methods

Methods 

*Design, Population, and Study Setting/Location* 

A retrospective cohort study was conducted for all intubated neonates admitted to the NICU at the Women’s Wellness and Research Center (WWRC), Hamad Medical Corporation (HMC) in Qatar, who received any form of mechanical ventilation. The study covered all UE events in the NICU over four years, from January 1, 2018, to December 31, 2021.

WWRC in Qatar, the largest tertiary maternity hospital in the region, boasts a 120-bed level 3 NICU. During the study period, the hospital recorded nearly 70,000 deliveries, leading to approximately 9,600 NICU admissions, including 507 EP neonates.

The study received approval from the Institutional Review Board (IRB) of the Medical Research Center (MRC), with the approval number MRC: 01-22-2588. Due to the retrospective nature of the study, informed consent was waived.

*Inclusion and Exclusion Criteria* 

All intubated EP neonates (gestational age (GA) less than 28 weeks) admitted to the NICU during the four-year study period were included, except for those who died before experiencing a UE event. For outcome analysis, the comparison group consisted of a matched cohort of EP neonates with available outcome data, including neurodevelopmental assessments at 18-22 months of postmenstrual age (PMA), extracted from a pool of 507 EP neonates born during the same period. All matched EP neonates had not experienced UE. We excluded 292 EP neonates who lacked neurodevelopmental outcome data.

*Data Collection* 

Data on UE events were collected from electronic charts and nursing and respiratory root cause analysis audits of UE events at WWRC/HMC. These audits were introduced as part of the care improvement bundle for intubated neonates and were collected immediately following UE events. The care improvement bundle included educational workshops to raise awareness among all staff about uniform methods for taping and securing ETTs, utilizing headgear to minimize sudden head movements, engaging a physiotherapist for proper head positioning and turning, adjusting ETT taping as needed, regular checks on ETT fixation level and security, prioritizing non-medication-based sedation and comfort strategies, optimizing bed setup for patient comfort including nesting and positioning, promoting the use of non-invasive respiratory support and introducing less invasive surfactant delivery techniques to reduce the need for intubation, scheduling linen changes and patient bathing to the evening shift instead of at midnight, implementing physician orders for minimal patient handling, placing high alert notices at the bedsides of all ventilated neonates, and employing a daily monitoring form to record all pertinent incidents and observations [1,13,17,22].

Collected data included the GA in weeks, sex, birth weight (BW), delivery mode, underlying respiratory diagnosis, date and time of intubation, mode, and pressures of mechanical ventilation (MV), chest X-ray findings, the fraction of inspired oxygen (FiO2), sedation dose and start date, and other medications. Sedation was administered only when indicated, such as for significant hypoxemia with evidence of pulmonary hypertension, high-frequency ventilation, or during invasive procedures like chest tube placement. Our protocol involves using morphine sulfate at a dosage of 20-30 micrograms per kilogram per hour for a short duration.

Additionally, data such as endotracheal tube (ETT) size, use of steroids, date and time of UE event, post-extubation condition, lowest heart rate (HR) and oxygen saturation, blood gas results, use of positive pressure ventilation, mode of respiratory support post-UE, date and time of reintubation, and any resuscitation measures (saline or epinephrine bolus received, and chest compressions), were collected.

Outcome Measures 

The primary outcome of the study was the UE incidence rate in EP neonates, defined as the number of UE events per 100 ventilator days. Data on both the total number of ventilator days and the total number of UE events were reported, facilitating the calculation of the combined UE incidence rate each month.

The secondary outcomes of the study focused on identifying risk factors and outcomes associated with UE. We collected data on patient demographics, clinical characteristics, event specifics, and related interventions, such as the need for resuscitation, reintubation, or modifications in respiratory support. Outcome measures included bronchopulmonary dysplasia (BPD) at 36 weeks: No BPD for no respiratory support, grade 1 for ≤2 LPM nasal cannula, grade 2 for >2 LPM or non-invasive support, and grade 3 for invasive ventilation [23]; ventilator-associated pneumonia (VAP), defined per CDC criteria: new or progressive infiltrates on chest X-ray, along with clinical signs such as fever, leukocytosis, purulent secretions, and worsening respiratory status in a mechanically ventilated infant [24]; culture-proven sepsis; intraventricular hemorrhage (IVH), classified per the Papile system: grade I (germinal matrix hemorrhage), grade II (IVH without ventricular dilation), grade III (IVH with ventricular dilation), and grade IV (IVH with parenchymal involvement) [25]; necrotizing enterocolitis (NEC), classified using the modified Bell’s staging: stage I (suspected NEC), stage II (proven NEC with mild to moderate symptoms), and stage III (advanced NEC with severe systemic illness) [26]; patent ductus arteriosus (PDA) defined as hemodynamically significant PDA requiring intervention; pulmonary hemorrhage; retinopathy of prematurity (ROP); periventricular leukomalacia (PVL); and neurodevelopmental outcomes. Basic clinical characteristics and outcome measures were compared to a matched control group of EP neonates born in the same period, with available data on outcomes, including neurodevelopmental assessments.

Neurodevelopmental assessments were conducted on neonates between 18 and 22 months of corrected age using the Bayley Infant Neurodevelopmental Screener (BINS III). Neonates were categorized based on their performance in critical developmental domains (cognitive, receptive, expressive, and neurological, including fine and gross motor skills).

*Statistical Analysis* 

Categorical data were presented as frequency and percentage for participant characteristics and analyzed using the Chi-square or Fisher’s exact test. Continuous variables with normal distribution were represented as mean ± SD, while non-parametric data were presented as median and interquartile range (IQR). The Student’s t-test was used to compare parametric variables, and the Mann-Whitney U test was used for non-parametric variables. Statistical significance was set at a two-tailed p-value of < 0.05. For baseline clinical characteristics and outcome analysis, propensity score matching (PSM) was used in a 1:4 ratio with a caliper value of 0.2, considering seven key baseline variables: BW, GA, gender, surfactant use, any intubation, FiO₂ levels >0.4, or any ventilator support during the first week. All statistical analyses were conducted using SPSS Statistics version 29.0.

## Results

We recorded 25 UE events in our cohort of EP neonates over the study period. Among 507 EP neonates, there were a total of 5,668 invasive ventilation days. The incidence of UE events was 0.44 per 100 ventilation days over the four years, ranging from 0.60 in 2018 to 0.27 in 2020.

Neonatal demographics and clinical characteristics 

Table 1 summarizes the baseline clinical characteristics of intubated neonates. Over the four-year study period, a total of 54 UE events were reported in the NICU, of which 25 occurred in EP neonates, with a median BW of 740 grams (IQR: 670-905) and a median GA of 25.0 weeks (IQR: 24.0-26.0). Males accounted for 64% of the cases. The primary diagnoses at the time of admission were respiratory distress syndrome (RDS) in 72.0%, bronchopulmonary dysplasia (BPD) in 20.0%, and pneumothorax in 8.0%.

**Table 1:** Neonatal demographics and clinical characteristics of all infants with unplanned extubation. Data are presented as number (%) or median (IQR). RDS: Respiratory distress syndrome; BPD: Bronchopulmonary dysplasia; HIE: Hypoxic-ischemic encephalopathy; ETT: Endotracheal tube; T2: Second thoracic vertebra; CXR: Chest X-ray; FiO₂: Fraction of inspired oxygen. *Highest within 24 hours.

Variable	Variable	N=25
Birth weight (grams), median (IQR)		740.0 (670.0, 905.0)
Gestational age (weeks), median (IQR)		25.0 (24.0-26.0)
Gender, n (%)	Males	16.0 (64.0%)
Diagnosis, any, n (%)	RDS	18.0 (72.0%)
	BPD	5.0 (20.0%)
	Pneumothorax	2.0 (8.0%)
Weight at the time of UE event (grams), median (IQR)		850.0 (730.0,1375.0)
Time of UE, median (IQR)		1:20 PM (8:50 AM,5:40 PM)
Postnatal age at the time of UE event (days), median (IQR)		15.0 (3.5-37.0)
Ventilation mode, n (%)	Conventional	19.0 (76.0%)
	High Frequency	6.0 (24.0%)
Sedation, n (%)	Yes	5.0 (20.0%)
ETT size (mm), n (%)	2.5	16.0 (64.0%)
	3.0	3.0 (12.0%)
	3.5	4.0 (16.0%)
	4.0	2.0 (8.0%)
ETT (Thoracic vertebral level) on the last CXR, median (IQR)		2.0 (2.0,2.75)
FiO_2_* (%), median (IQR)		52.0 (30.0,75.0)

At the time of the UE episode, the neonates had a median weight of 850 grams (IQR: 730-1375), and the event occurred at a median postnatal age of 15.0 days (IQR: 3.5-37.0), typically in the afternoon around a median of 1:20 PM (IQR: 8:50 AM-5:40 PM). Of the 25 cases, 76.0% were on conventional ventilation, while 24.0% were on high-frequency ventilation, with a median fraction of inspired oxygen (FiO₂) of 52% (IQR: 30.0-75.0).

About 20.0% of neonates received sedation during the event. The endotracheal tube (ETT) sizes used were as follows: 2.5 in 64.0% of the neonates, 3.0 in 12.0%, 3.5 in 16.0%, and 4.0 in 8.0%. The median ETT tip position on the most recent CXR was at the second thoracic vertebra (T2) (IQR: 2.0-2.75).

The clinical characteristics of all infants during the UE event are presented in Table 2. Various events related to UE were documented, including agitation (20%), handling of the ETT (20%), during routine patient care (24%), high ETT position (12%), procedures (12%), and vomiting (8%).

**Table 2:** Clinical characteristics of unplanned extubation events of all infants with unplanned extubation. Data are presented as number (%) or median (IQR). UE: Unplanned extubation; iNO: Inhaled Nitric Oxide; FiO2: Fraction of inspired oxygen; CXR: Chest X-ray; PPV: Positive pressure ventilation. ^*^Highest within 24 hours ^**^Three additional neonates were intubated within 72 hours. ^&^Among those who were reintubated. ^$^Defined as the increase in FiO_2_ requirement by ≥25%, initiation of inhaled Nitric Oxide, or changes to ventilatory mode or setting. Missing data: CXR repeat day (n=4).

Variable	Event	N=25
Event during UE, n(%)	No record	1.0 (4.0)
	Agitation	5.0 (20.0)
	Handling ETT	5.0 (20.0)
	Routine patient care	6.0 (24.0)
	High ETT	3.0 (12.0)
	Procedure	3.0 (12.0)
	Vomiting	2.0 (8.0)
FiO_2_ after UE^*^, median (IQR)		95.0 (40.0,100.0)
Reintubated^**^, n(%)	Yes	22.0 (88.0)
No of reintubation trial^&^ , n (%)	1	16.0 (72.7)
	2	5.0 (22.7)
	3	1.0 (4.5)
FiO_2_ change from baseline value (24h), n(%)	Less than baseline	2.0 (8.0)
	No change	6.0 (24.0)
	Increased by ≤25%	5.0 (20.0)
	Increased by 26-49%	8.0 (32.0)
	Increased by ≥50%	4.0 (16.0)
Started on iNO, n(%)	Yes	5.0 (20.0)
Existing iNO use change, n(%)	Yes	7.0 (28.0)
Ventilator mode change, n(%)	Yes	2.0 (8.0)
Any respiratory escalation^$^, n(%)	Yes	14.0 (56.0)
CXR repeated, n(%)	Yes	24.0 (96.0)
CXR repeat day, n(%)	Same day	13.0 (54.2)
	One-day later	8.0 (33.3)
	>2 days later	3.0 (12.5)
CXR change, n(%)	No significant change	15.0 (62.5)
	Pneumothorax	2.0 (8.3)
	Opacification/Collapse	6.0 (25.0)
	Hyperinflation	1.0 (4.2)
Received PPV, n(%)	Yes	16.0 (64.0)
Received chest compression, n(%)	Yes	1.0 (4.0)
Received epinephrine, n(%)	Yes	1.0 (4.0)

Reintubation was required for 88% of the neonates, with 72.7% requiring one reintubation attempt, 22.7% two attempts, and 4.5% three attempts. Following UE, FiO₂ decreased from baseline in 8% of cases, remained unchanged in 24% of patients, and increased from the baseline by ≤ 25%, 26-49%, and ≥50% in 20%, 32%, and 16% of patients, respectively. Inhaled nitric oxide (iNO) was initiated in 20% of neonates, and existing iNO use was escalated in 28%.

Respiratory support was escalated in 56% of cases. Chest X-rays (CXR) were repeated in 96% of neonates, with 54.2% performed on the same day, 33.3% one day later, and 12.5% more than two days later. CXR changes included no significant findings in 62.5%, pneumothorax in 8.3%, opacification/collapse in 25%, and hyperinflation in 4.2%. Positive pressure ventilation (PPV) was required for 64% of neonates post-UE.

Table 3 shows the clinical characteristics of neonates before and after employing propensity score matching (PSM). PSM was applied to create comparable groups between EP neonates with UE and those without UE, ensuring that differences in outcomes could be more accurately attributed to the extubation events rather than to underlying disparities in clinical characteristics. The matching was performed on a cohort of 216 EP infants, with available outcome data, using eight key baseline variables: BW, GA, gender, surfactant use, intubation status, any FiO₂ levels>0.4, and ventilator needs during the first week of life beyond the initial 48 hours from birth.

**Table 3:** Comparison of baseline characteristics between extremely preterm infants with and without unplanned extubation using propensity score matching. Data are expressed as No. (%), Mean (standard deviation), or Median (interquartile range, IQR). Comparisons between groups were performed using the Chi-square (χ²) test or Fisher’s exact test for categorical variables, the Student’s t-test for normally distributed continuous variables, and the Mann-Whitney U test for non-normally distributed continuous variables. GA: Gestational age; BW: Birth weight; SGA: Small for gestational age; FiO_2:_ Fraction of inspired Oxygen. Missing values: Pulmonary hemorrhage (n=9), Apgar scores (n=10).

Variable	Before Matching				After Matching			
Outcome	UE (N=25)	No UE (N=216)	X2/Z/t value	P-value	UE (N=25)	No UE (N=75)	X2/Z/t value	P-value
GA, weeks (mean ± SD)	25.0±1.2	25.6±1.2	-2.088	0.037	25.0±1.2	24.9±1.8	-0.633	0.528
BW, g (mean ± SD)	788.8±162.6	850.4±182.5	1.615	0.108	788.8±162.5	786.3±171.0	-0.063	0.950
Male gender, No. (%)	16.0 (64.0)	119.0 (55.1)	0.722	0.396	16.0 (64.0)	41.0 (54.7)	0.666	0.414
SGA, No. (%)	2.0 (8.0)	12.0 (5.6)	0.245	0.644	2.0 (8.0)	3.0 (4.0)	0.632	0.596
APGAR score 1 min<7, No. (%)	18.0 (72.0)	147.0 (71.4)	0.004	0.947	18.0 (72.0)	55.0 (73.3)	0.017	0.897
APGAR score 5 min<7, No. (%)	4.0 (16.0)	43.0 (20.9)	0.327	0.568	4.0 (16.0)	18.0 (24.0)	0.699	0.403
Vaginal birth, No. (%)	10.0 (40.0)	87.0 (40.3)	0.001	0.979	10.0 (40.0)	38.0 (50.7)	0.855	0.355
Surfactant, No. (%)	25.0 (100)	178.0 (82.4)	5.221	0.018	25.0 (100.0)	73.0 (97.3)	0.680	0.409
Antenatal Steroids, No. (%)	23.0 (92.0)	182.0 (84.3)	1.057	0.389	23.0 (92.0)	62.0 (82.7)	1.281	0.258
Endotracheal intubation (first week), No. (%)	24.0 (96.0)	186.0 (86.1)	1.955	0.217	24.0 (96.0)	71.0 (94.7)	0.070	1.000
Pulmonary hemorrhage No. (%)	3.0 (12.0)	12.0 (5.8)	1.419	0.234	3.0 (12.0)	7.0 (9.3)	0.148	0.708
Pneumothorax No. (%)	2.0 (8.0)	9.0 (4.2)	0.756	0.319	2.0 (8.0)	4.0 (5.3)	0.236	1.000
Any FiO2 ≥ 0.4 (first week), No (%)	13.0 (52.2)	71.0 (33.2)	3.479	0.062	13.0 (52.0)	34.0 (45.3)	0.335	0.563
Any invasive ventilation (first week), No (%)	23.0 (92.0)	179.0 (82.9)	1.377	0.388	23.0 (92.0)	72.0 (96.0)	0.632	0.596

After PSM, a final cohort of 25 EP infants with UE and 75 without UE was obtained, resulting in balanced groups for BW, GA, gender, and other critical variables. Before matching, significant differences were noted, such as lower GA (25.0 vs. 25.6 weeks, p = 0.037) and higher use of surfactant in the UE group (100% vs. 82.4%, p = 0.018). However, after matching, none of the baseline variables, including BW (788.8 vs. 786.3 g, p = 0.950) and GA (25.0 vs. 24.9 weeks, p = 0.528), showed significant differences, indicating that the matching procedure successfully balanced the groups.

In the comparison of outcomes between EP infants with UE and those without UE (Table 4), significant differences were observed both before and after PSM. Before matching, the UE group had a median of 25.0 days on mechanical ventilation compared to 3.5 days in the no-UE group (Z = -4.534, p < 0.001). The incidence of BPD was markedly higher in the UE group compared to the no-UE group (91.3% vs. 43.5%, χ² = 18.716, p < 0.001 before matching, and 91.3% vs. 59.6%, χ² = 7.643, p = 0.006 after matching). Severe BPD was also significantly more prevalent in the UE group (34.8% vs. 8.8%, χ² = 8.147, p = 0.008). After matching, the UE group had nonsignificantly longer ventilation days (25.0 vs. 16.0 days, Z = -1.700, p = 0.089).

**Table 4:** Comparison of outcomes between extremely preterm infants with unplanned extubation and those without unplanned extubation using propensity score matching. Data are expressed as No. (%), Mean (SD), or Median (IQR). Comparisons between groups were performed using the Chi-square (χ²) test or Fisher’s exact test for categorical variables, the Student’s t-test for normally distributed continuous variables, and the Mann-Whitney U test for non-normally distributed continuous variables. BPD: Bronchopulmonary dysplasia; VAP: Ventilator-associated pneumonia; IVH: Intraventricular hemorrhage; PVL: Periventricular leukomalacia; ROP: Retinopathy of prematurity; PDA: Patent ductus arteriosus; NEC: Necrotizing enterocolitis; PMA: Postmenstrual age. ^*^Reference: Jensen EA et al., 2019 [23]. ^#^Reference: Centers for Disease Control and Prevention, 2024 [24]. ^$^Reference: Papile LA et al., 1978 [25]. ^&^Reference: Walsh MC et al., 1986 [26]. Missing Values: BPD (n=34, due to either death or loss to follow-up before 36 weeks postmenstrual age); EOS (n=36); IVH/PVL (n=7); ROP (n=22); Neurodevelopmental assessment (n=42, due to either death or loss to follow-up before 36 weeks postmenstrual age); Hospital stays and discharge PMA (n=52).

Outcome	Before Matching				After Matching			
Outcome	UE (N=25)	No UE (N=216)	X2/Z/t value	P	UE (N=25)	No UE (N=75)	X2/Z/t value	P
Invasive ventilation days, (Median, IQR)	25.0(10.5, 96.5)	3.5(0.3, 19.0)	-4.534	<0.001	25.0(10.5, 96.5)	16.0(6.0, 32.0)	-1.700	0.089
Death, No. (%)	5.0(20.0)	32.0(14.8)	0.450	0.557	5.0(20.0)	18.0(24.0)	0.169	0.681
BPD*, No. (%)	21.0(91.3)	80.0(43.5)	18.716	<0.001	21.0(91.3)	34.0(59.6)	7.643	0.006
Severe BPD*, No. (%)	8.0(34.0)	8.0(4.3)	26.552	<0.001	8.0(34.8)	5.0(8.8)	8.147	0.008
Severe BPD* or death No. (%)	11.0(44.0)	40.0(18.6)	8.631	0.008	11.0(44.0)	23.0(30.7)	1.485	0.223
Postnatal steroids, No. (%)	18.0(72.0)	25.0(11.6)	55.812	<0.001	18.0(72.0)	14(18.7)	24.510	<0.001
Proven early sepsis, No. (%)	0.0(0.0)	3.0(1.4)	0.127	1.000	0.0(0.0)	1.0(2.0)	0.337	1.000
Proven Late sepsis, No. (%)	4.0(16.0)	36.0(16.7)	0.007	1.000	4.0(16.0)	23.0(30.7)	2.046	0.153
VAP^#^ No. (%)	10.0(40.0)	40.0(18.5)	6.288	0.012	10.0(40.0)	28.0(37.3)	0.057	0.812
IVH^$^, No. (%)	9.0(36.0)	54.0(25.7)	1.205	0.272	9.0(36.0)	24.0(32.4)	0.107	0.744
Severe IVH (grades III&IV), No. (%)	1.0(4.0)	10.0(4.8)	0.029	1.000	1.0(4.0)	7.0(9.5)	0.750	0.387
PVL, No. (%)	1.0(4.0)	6.0(2.9)	0.098	0.754	1.0(4.0)	2.0(2.7)	0.107	1.000
ROP, No. (%)	16.0(69.6)	110.0(56.9)	1.460	0.227	16.0(69.6)	60.0(78.1)	0.677	0.411
ROP, stages ≥3, No. (%)	7.0(30.4)	33.0(16.9)	2.507	0.113	7.0(30.0)	20.0(31.3)	0.005	0.942
ROP requiring treatment, No. (%)	1.0(4.3)	18.0(9.2%)	0.617	0.701	1.0(4.3)	9.0(14.1)	1.570	0.210
PDA, medically treated, No. (%)	7.0(28.0)	66.0(30.6)	0.069	0.792	7.0(28.0)	33.0(44.0)	2.000	0.157
PDA, device closure, No. (%)	2.0(8.0)	10.0(4.6)	0.538	0.359	2.0(8.0)	8.0(10.7)	0.700	0.148
NEC^&^, medically treated, No. (%)	1.0(4.0)	15.0(7.2)	0.366	1.000	1.0(4.0)	14.0(18.7)	1.894	0.107
NEC^&^, surgically treated No. (%)	1.0(4.0)	5.0(2.4)	0.222	0.499	1.0(4.0)	5.0(6.7)	0.236	1.000
Cognitive delay, No. (%)	4.0(20.0)	13.0 (7.3)	3.736	0.075	4.0(20.0)	6.0(10.7)	1.112	0.292
Receptive delay, No. (%)	10.0(50.0)	27.0(15.1)	14.490	<0.001	10.0(50.0)	11.0(19.5)	6.792	0.009
Expressive delay, No. (%)	9.0(45.0)	51.0(28.5)	2.328	0.127	9.0(45.0)	19.0(33.9)	0.776	0.378
Neurologic delay, No. (%)	10.0(50.0)	37.0(20.7)	8.578	0.009	10.0(50.0)	21.0(37.5)	0.953	0.329
Developmental delay high-risk, No (%)	7.0(35.0)	21.0 (11.7)	8.056	0.011	7.0(35.0)	12.0 (21.4)	1.448	0.229
Hospital stay, days (Median, IQR)	127.0(112.0,183.2)	93.0(71.7,126.2)	-3.975	<0.001	127.0(112.0,183.2)	101.0(90.0,139.5)	-2.574	0.010
Discharge PMA, weeks (Median, IQR)	42.4(40.0, 49.7)	38.7(36.4, 43.0)	-2.569	0.010	42.8(41.1, 50.4)	40.1(37.3, 46.6)	-2.754	0.006

The need for postnatal steroids was substantially higher in the UE group, both before and after matching (72.0% vs. 11.6%, χ² = 55.812, p < 0.001 before matching, and 72.0% vs. 18.7%, χ² = 24.510, p < 0.001 after matching). Although the combined outcome of severe BPD or death was higher in the UE group before matching (44.0% vs. 18.6%, χ² = 8.631, p = 0.008), the difference was not statistically significant post-matching (44.0% vs. 30.7%, χ² = 1.485, p = 0.223). No significant differences were observed in complications such as late-onset sepsis, NEC, ROP, PDA, VAP, any brain injury, or severe IVH, either before or after matching.

Additionally, neurodevelopmental delays were significantly more common in the UE group after matching, particularly for receptive delay (50.0% vs. 19.5%, χ² = 6.792, p = 0.009). Patients with UE had a median longer hospital stay of 127.0 days compared to 101.0 days for those without UE events (Z = -2.574, p = 0.010), and a higher median discharge postmenstrual age (PMA) of 42.4 weeks versus 40.1 weeks (Z = -2.754, p = 0.006).

## Discussion

This study aimed to explore the incidence of UE, identify associated neonatal risk factors, and assess health outcomes following the implementation of a comprehensive care improvement bundle. Over four years following the implementation of the improvement bundle, we described factors surrounding UE and outcomes in our NICU. UE was generally associated with several events, such as agitation, handling of the ETT, routine patient care, high ETT position, vomiting, and procedures. Additionally, it was associated with reintubation trials and increased requirements for respiratory support. Repeated chest X-rays were necessary and identified significant parenchymal changes, requiring significant respiratory escalation and an increased need for PPV.

Before implementing the improvement care bundle, the unit lacked standardized procedures or assessments regarding ETT placement. Nurses relied on their own experience to evaluate the ETT, considering factors such as agitation, loose or wet taping, and bedside procedures. They seldom referred to chest X-rays to verify the position of the ETT tip. Nurses recorded basic patient information during a UE and reintubated the infant [12]. However, Cho JE and Yeo JH identified several UE risk factors, including the administration of sedatives or analgesics, failure to re-fixate the ETT, increased incidence of suctioning, working night shifts (from 5 p.m. to 8 a.m.) on weekdays or weekends, and a high nurse-patient ratio [14].

Various clinical outcomes were observed in relation to UE events. However, PSM was performed to minimize selection bias by creating comparable groups of EP neonates with and without UE. Based on our results after matching, we found that several risk factors were associated with an increased likelihood of UE and subsequent complications. Specifically, there was a statistically significant association between UE and higher rates of BPD and extended hospital stays. Similar findings have demonstrated that even in the absence of a UE event, the act of reintubation has been linked to higher rates of BPD and mortality [15]. Prior investigations have documented varying rates of reintubation within 24 hours following UE, ranging from 25% to 75% [8,22,27,28], which aligns with the findings reported by Pavlek LR et al. [29] and Kambestad KK et al. concerning immediate and total reintubation rates [3]. Moreover, Klugman D et al. conducted a study where they found that the majority of UE events require reintubation within one hour, accounting for an average of 120 out of 206 events/month (58.3%) compared to the events without reintubation, i.e., 78 out of 206 events/month (37.9%) [30].

UE has been associated with both short- and long-term morbidities. For instance, 10% of cases involved airway trauma requiring reintubation, while 42% needed multiple attempts for successful reintubation, and 39% experienced an increase in baseline oxygen requirements. Patients with BPD showed higher odds of experiencing repeated UE events, which were linked to elevated rates of tracheostomy and prolonged hospital stays. The higher incidence of repeated UEs in patients with BPD is likely due to the extended duration of intubation, increasing the chances for UE incidents to occur [28]. Among infants born at less than 29 weeks of gestation, reintubation following a UE event was associated with prolonged mechanical ventilation and a higher risk of developing BPD [14]. Pavlek LR et al. also demonstrated an association between BPD and UE events [27].

Additionally, findings by Kambestad KK et al. revealed that the substantial rise in the required post-UE airway pressure and FiO₂ requirements suggests that UE led to worsening respiratory status for many newborns, necessitating urgent reintubation [3]. In pediatric cardiac ICUs, the risks of ventilator-associated pneumonia (VAP) were found to be at least three times higher in patients who experienced UE [31]. In our study, although the VAP rate was higher in neonates with UE before matching, the difference was not statistically significant after matching, indicating that other baseline variables may have confounded the initial association. Notably, 50% of the VAP episodes in neonates with UE occurred within seven days of the event, highlighting a potential temporal link between UE and subsequent VAP development.

Cardiovascular collapse emerged as the study's most significant outcome of UE, aligning with prior research findings [3,19,30,32]. Klugman D et al. reported an average of 3.9% cardiovascular collapse associated with UE, i.e., 8 out of 206 monthly events [30]. Similarly, two earlier studies by Hatch LD et al. and Horimoto Y et al. reported bradycardia in 46% and 32% of UE events, respectively, with corresponding rates of CPR of 12% and 6% [6,33]. The risk of sepsis is substantially higher in individuals requiring CPR [3]. Furthermore, another investigation observed a correlation between the nursing overtime ratio in the NICU and an increased occurrence of UE events. Dominick CL et al. found that nursing care was one of the contributing factors in 18% of UE events [34]. In our cohort, 64% of babies with UE received PPV, but only one baby required chest compression and epinephrine administration.

Evidence supports that adherence to a care bundle can reduce UE rates. Merkel L et al. reported a significant decrease in UE events through developing a comprehensive bundle and implementing quality improvement (QI) initiatives by plan-do-study-act cycles at a single-center tertiary NICU [1]. Klugman D et al.'s multi-center QI initiative documented a 24.1% reduction in UE events in 43 children’s hospitals, decreasing from 1.135 to 0.862 UEs per 100 ventilator days. Notable improvements were seen in both pediatric and neonatal ICU settings. The study also highlighted a 36.6% reduction in severe outcomes, including cardiovascular collapse, which dropped from 0.041 to 0.026 UE events per 100 ventilator days [30].

In alignment with these findings, our study observed a significant reduction in UE incidence, from 0.73% to 0.32% per 100 ventilator days, over the four years from 2018 to 2021. This improvement followed the implementation of a bundled care approach for intubated infants on mechanical ventilation, highlighting the effectiveness of coordinated care interventions in reducing UE rates in critical care settings.

Several QI studies have successfully employed care bundles to decrease UE events in the NICU. Only a limited number of studies have specifically focused on neonates born at less than 29 weeks of gestation or evaluated BPD as a specific outcome [1,13,27,35]. Common strategies in these studies include using a standardized approach for securing the ETT based on anatomical reference points, ensuring two staff members are present during interventions or movements involving intubated neonates, and providing continuous staff training and routine reminders of prevention methods [1,12,35]. Moreover, Hu X et al. reported improved compliance with ETT securement measures, position records, and overall tube security in a follow-up audit. Compliance with care preparation records increased from 0% to 100%, adherence to standard care practices increased from 0% to 54.9%, and staff learning compliance improved from 66.7% to 100% [12].

Strength and limitations 

This study identifies clinical risk factors and adverse neonatal health outcomes associated with UE events in a cohort of EP neonates in a tertiary NICU center in Qatar. It emphasizes a population at the highest risk, particularly vulnerable to sudden cardiorespiratory decompensation and the need for reintubation. The study was designed to include matched controls, minimizing bias and allowing for a more accurate assessment of the true impact of UE on neonatal outcomes by balancing key baseline variables between the groups. Additionally, the inclusion of long-term neurodevelopmental outcomes is particularly important for EP infants, who are already at high risk for developmental impairments.

The study has several limitations that should be considered. It is a single-center study with a small sample size, which is attributed to the low occurrence of UE events. Therefore, the generalizability of our findings may be limited, and larger multicenter studies are necessary to validate the results. Being a retrospective study raises the possibility of limited or missing data. However, this concern is partially addressed because patients were identified at each UE event, and documentation was completed around the event.

The associations between UE and adverse outcomes should not be interpreted as causative. There may still be other confounding factors not accounted for in the matching process, which could complicate the interpretation of our findings. This highlights the need for careful interpretation of our results and suggests that future studies should aim to better define the relationships between UE and neonatal outcomes and explore how reducing UE impacts a broader range of neonatal health outcomes to better understand its clinical implications.

## Conclusions

Despite the observed reduction in UE rates in EP infants after implementing a care improvement bundle, EP infants with UE experienced significantly increased rates of BPD, higher use of postnatal steroids, and extended NICU stays. Additionally, these infants were found to have a higher risk of receptive neurodevelopmental delay, indicating that UE events are associated not only with respiratory complications but also with impaired neurodevelopmental outcomes in this vulnerable population.

These findings highlight the urgent need for strong prevention and management strategies for UE in NICUs. Identifying specific patient characteristics associated with higher UE rates can guide the development of targeted prevention strategies for high-risk neonates. NICU practices should prioritize reducing UE rates and the subsequent need for emergent reintubations by implementing care improvements tailored to address these specific challenges. Further research is also necessary to evaluate the direct impact of UE rate reduction on neonatal outcomes, particularly within comprehensive care plans.
